# Synthesis and Properties of CurNQ for the Theranostic Application in Ovarian Cancer Intervention

**DOI:** 10.3390/molecules25194471

**Published:** 2020-09-29

**Authors:** Lara G. Freidus, Pradeep Kumar, Thashree Marimuthu, Priyamvada Pradeep, Viness Pillay, Yahya E. Choonara

**Affiliations:** Wits Advanced Drug Delivery Platform Research Unit, Department of Pharmacy and Pharmacology, School of Therapeutic Sciences, Faculty of Health Sciences, University of the Witwatersrand, Johannesburg, 7 York Road, Parktown 2193, South Africa; lara.freidus@students.wits.ac.za (L.G.F.); pradeep.kumar@wits.ac.za (P.K.); thashree.marimuthu@wits.ac.za (T.M.); priyamvada.pradeep@wits.ac.za (P.P.); viness.pillay@wits.ac.za (V.P.)

**Keywords:** curcumin, lawsone, theranostics, pH responsivity, fluorescence, high content imaging

## Abstract

Synthesis of a novel theranostic molecule for targeted cancer intervention. A reaction between curcumin and lawsone was carried out to yield the novel curcumin naphthoquinone (CurNQ) molecule (2,2′-((((1E,3Z,6E)-3-hydroxy-5-oxohepta-1,3,6-triene-1,7-diyl) bis(2-methoxy-4,1-phenylene))bis(oxy))bis(naphthalene-1,4-dione). CurNQ’s structure was elucidated and was fully characterized. CurNQ was demonstrated to have pH specific solubility, its saturation solubility increased from 11.15 µM at pH 7.4 to 20.7 µM at pH 6.8. This pH responsivity allows for cancer targeting (Warburg effect). Moreover, CurNQ displayed intrinsic fluorescence, thus enabling imaging and detection applications. In vitro cytotoxicity assays demonstrated the chemotherapeutic properties of CurNQ as CurNQ reduced cell viability to below 50% in OVCAR-5 and SKOV3 ovarian cancer cell lines. CurNQ is a novel theranostic molecule for potential targeted cancer detection and treatment.

## 1. Introduction

Natural compounds provide a significant database of potential drug candidates and play a pivotal role in drug discovery [1,2]. Phytochemicals are naturally occurring compounds and have been used for centuries to treat and prevent many illnesses [3]. Several phytochemical compounds have been shown to have potent bioactive moieties with a few even showing promise as anticancer molecules [4] through the modulation of several biochemical pathways and processes associated with oncogenesis [5].

An interesting concept of late in anticancer interventions are theranostic systems (i.e., combining diagnostic and therapeutic elements in a single platform) in which chemically modified phytochemicals could play a significant role [6,7]. Theranostic agents are highly advanced systems that can function to monitor the accumulation of various compounds (including nanomedicines) at target sites, quantify triggered drug release, and detect malignant cells in order to deliver a chemotherapeutic agent to the targeted site [7,8]. The ability to simultaneously detect and treat cancer also reduces patient mortality as a result of early disease detection and immediate treatment [9].

Epithelial ovarian carcinoma (OC) is the most common type of OC, accounting for 90% of ovarian tumors. Symptoms of OC are detected late resulting in diagnosis at stages III and IV [10,11]. In addition, the highly metastatic nature of OC results in low patient survival with a mortality of approximately 60% [12]. A high rate of chemoresistance and relapse has also been associated with OC [13]. Therefore, newer interventions are urgently required to provide earlier detection of OC and provide a more targeted form of chemotherapy. The metabolism of a cancer cell is unique in that it undergoes aerobic glycolysis (the Warburg effect) [14]. This phenomenon can therefore be exploited to design a targeted theranostic system for the detection and treatment of OC.

In order to harness the benefits of utilizing a phytochemical in the design of a theranostic system for OC, curcumin (CUR) (diferuloylmethane), a natural polyphenol was selected based on multiple studies that reported on CUR to modulate signaling pathways in OC as an anticancer molecule [including the nuclear factor kappa-light-chain-enhancer of activated B cells (NF-κB) pathway, phosphatidylinositol 3-kinase/protein kinase B (PI3K/AKT) pathway, epidermal growth factor receptor (EGFR) pathway, nuclear factor erythroid 2-related factor 2 (Nrf2) pathway, Notch 1 pathway, the canonical Wnt pathway (Wnt/β-catenin) pathway, and the hypoxia-inducible factor 1 (HIF-1) pathway]. In addition, studies have demonstrated CUR to reduce proliferation and metastasis as well as induce apoptosis [15,16,17,18,19,20,21]. Specifically, in OC, lysophosphatidic acid (LPA) stimulates tumor development, progression, motility and metastasis when over-expressed and has been detected in 98% of OC tumors. CUR inhibits LPA, thus reducing OC cell motility [12,22]. In addition, studies have shown that CUR can inhibit NF-κB and thereby reducing inflammation [4,23,24]. Other studies have reported CUR to induce reactive oxygen species (ROS) production that stimulates the expression of DR5 on the cell surface resulting in tumor necrosis factor-related apoptosis-inducing ligand (TRAIL) mediated apoptosis as well as sensitizing cells to TRAIL via inhibition of NF-κB [25,26]. Furthermore, various researchers have demonstrated CUR to disrupt the PI3K/Akt pathway which is involved in OC development [19,27,28]. CUR is therefore a unique phytochemical that has been shown to interact with multiple signaling pathways in OC and to function as a therapeutic agent.

In addition, its anticancer properties, CUR has fluorescent properties [29,30]. However, the fluorescent lifetime is short (degraded within 30 min with a low quantum yield) at physiological pH [31,32]. This impedes its suitability to be also used as an in vivo imaging agent as part of a new theranostic system for OC. Moreover, CUR readily degrades in a variety of standard conditions, has negligible aqueous solubility and bioavailability, undergoes rapid metabolism, has poor photostability and has many pan-assay interference compound (PAINS) characteristics [33,34]. Numerous studies have focused on improving the solubility and bioavailability of CUR, but none have been successful in resolving the many stability and bioavailability issues faced in vivo. Therefore, in addition to utilizing CUR for the design of a new theranostic system in OC, this study aimed to improve the biopharmaceutical properties of CUR to further exploit the purported anticancer therapeutic properties of the phytochemical by synthesizing a novel therapeutically stable molecule.

In order to achieve this, another natural molecule lawsone (2-hydroxy-1, 4-naphthoquinone) (a chemical constituent of the henna plant) was also selected based on its demonstratable anticancer activity [35,36]. The 1,4-naphthoquinone pharmacophore has been at the center of numerous studies and has reported biological activity via a dianion species or radical anion [36]. Quinone moieties are present in several anticancer drugs including daunorubicin, doxorubicin and mitoxantrone [37] and many derivatives synthesized from 2-hydroxy-1,4-naphthoquinone have been demonstrated to have anticancer activity [38] resulting from redox cycling, arylation, intercalation, the induction of DNA strand breaks, the generation of free radicals and alkylation via quinone methide formation [38]. The anticancer activity of lawsone (1,4 naphthoquinone) has been attributed to its interaction with various signaling pathways via inducing the rapid formation of ROS which induces DNA damage and leads to cell death [39]. Quinones are widely known for their fluorescent properties and many quinone derivatives are used as dyes and fluorescent labels [40]. Thus, lawsone exhibits inherent fluorescent properties owing to its structure, making it possible to be incorporated within a new anti-cancer theranostic system. 

Therefore, this study focuses on combining an imaging and a therapeutic agent into a single platform by synthesizing a novel theranostic compound that combines CUR and lawsone in order to take advantage of the anticancer properties [18,19,21,22,27,41] (therapeutic) as well as the fluorescent properties, particularly of lawsone [40,42] (diagnostic). Although both phytochemicals have independently been shown to elicit anticancer activity, each have several limiting factors to advance their clinical application. These limiting factors include molecular instability, rapid degradation and poor bioavailability; this work also aims to overcome some of these limitations by the synthesis of a stable molecule with appropriate bioavailability, targeting capabilities and innate fluorescence. In addition, to facilitate the new theranostic system to function as an anticancer passive targeting system, the new combined molecule has been designed to have pH-responsivity based on the known acidification of the extracellular matrix (ECM) during malignant transformation [14]. A Williamson ether synthesis reaction between CUR and lawsone was used as the chemical synthesis route to produce the novel theranostic compound termed curcumin naphthoquinone (CurNQ) [2,2′-((((1E,3Z,6E)-3-hydroxy-5-oxohepta-1,3,6-triene-1,7-diyl)bis(2-methoxy-4,1-phenylene))bis(oxy))bis(naphthalene-1,4-dione)]. 

## 2. Results

**Figure 1:**
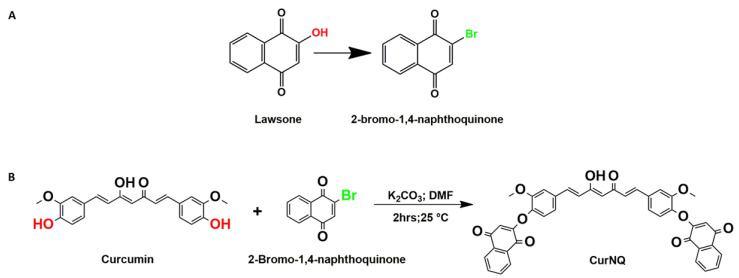
Synthesis of curcumin naphthoquinone (CurNQ). (**A**) Structural comparison between lawsone and 2-bromo-1,4-naphthoquinone (BrNQ). (**B**) Reaction scheme for CurNQ synthesis.

In order to react CUR and lawsone, the hydroxyl moiety of lawsone was required to be replaced with a chemically reactive site such as bromine. To save time, commercially available 2-bromo-1,4-naphthoquinone (BrNQ) was used, thereby reducing the steps required prior to the synthesis of the desired molecule. The desired molecule was synthesized through a Williamson ether synthesis reaction between CUR and BrNQ (Figure 1). Subsequent to synthesis and purification through silica column chromatography (0.015–0.040 mm) in a 30% ethyl acetate/hexane solution, the purified product was dried under reduced pressure and the product of the reaction was sent for ^1^H and ^13^C NMR and HPLC high resolution mass spectrometry (HRMS)-electrospray ionization (ESI) to confirm the synthesis of the desired molecule. 

**Figure 2:**
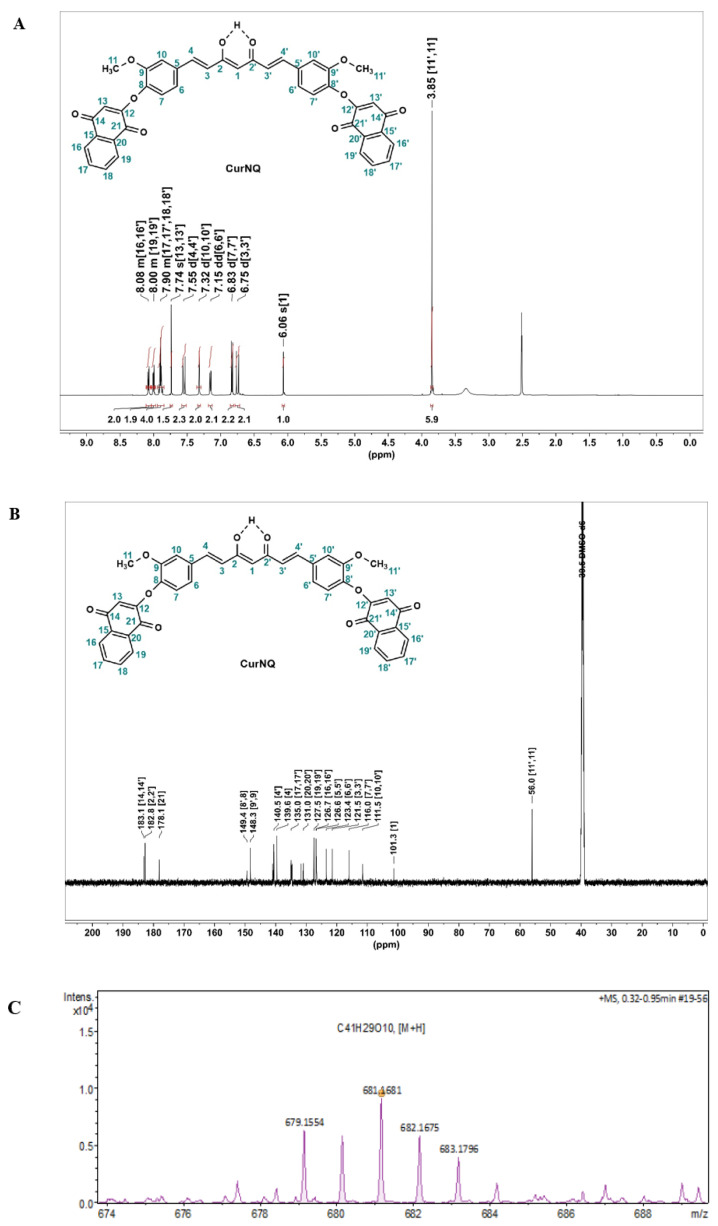
Spectral analysis of synthesized molecule (**A**) ^1^H NMR of CurNQ in deuterated dimethyl sulfoxide (d-DMSO)^1^H NMR (500 MHz, DMSO-d 6) δ 8.11–8.04 (m, 2H), 8.04–7.97 (m, 2H), 7.94–7.85 (m, 4H), 7.74 (s, 2H), 7.55(d, *J* = 15.8 Hz, 2H), 7.32 (d, *J* = 2.0 Hz, 2H), 7.15 (dd, *J* = 8.3, 2.0 Hz, 2H), 6.83 (d, *J* = 8.2 Hz, 2H), 6.75 (d, *J* =15.8 Hz, 2H), 6.06 (s, 1H), 3.85 (s, 6H). (**B**) ^13^C NMR of CurNQ in d-DMSO δ 183.1 (C=O), 182.8 (C=O), 178.1(C=O), 149.4, 148.3, 141.0, 140.5, 139.6, 134.96, 134.7, 131.7, 131.0, 127.5, 126.7, 126.6, 123.4, 121.5, 116.0, 111.5, 101.3, 56.0 (OCH_3_). (**C**) HRMS-ESI spectrum of CurNQ.

^1^H and ^13^C NMR spectra were obtained for the novel molecule (Figure 2). In the ^1^H NMR (Figure 2A). The signal at 3.85 ppm, which integrates to six protons, was indicative of two methoxy groups (protons H11 and H11′) on the CUR backbone. These methoxy groups have a similar spatial environment and, because of this, the peaks for both appear at the same chemical shift. Moreover, doublets at 7.55 (d, *J* = 15.8 Hz, 2H), and 6.75 (d, *J* = 15.8 Hz, 2H) were characteristic of the alkenyl protons on CUR backbone. New multiplets centered around δ 8.08 (m, 2H), 8.00 (m, 2H), and 7.90 (m, 4H), were assigned to the aromatic phenyl protons and a singlet at 7.74 ppm was ascribed to the cyclic alkenyl proton, which are indicative of the successful derivatization of CUR with BrNQ. 

In the ^13^C NMR (Figure 2B), two significant peaks appear at 183.1 and 178.1 ppm; these were assigned to the carbonyl (C=O, ketones) at C14,14′ and C21,21′, respectively. Moreover, the keto/enol carbon present at 182.8 ppm represents the distinctive keto/enol system of CUR. In the mass spectrum (Figure 2C), a peak at 681.176 corresponded with the exact mass of CurNQ with the addition of one hydrogen atom [M + H]^+^. This analysis gives a complementary characterization and is also indicative of the successful synthesis and isolation of CurNQ.

**Figure 3:**
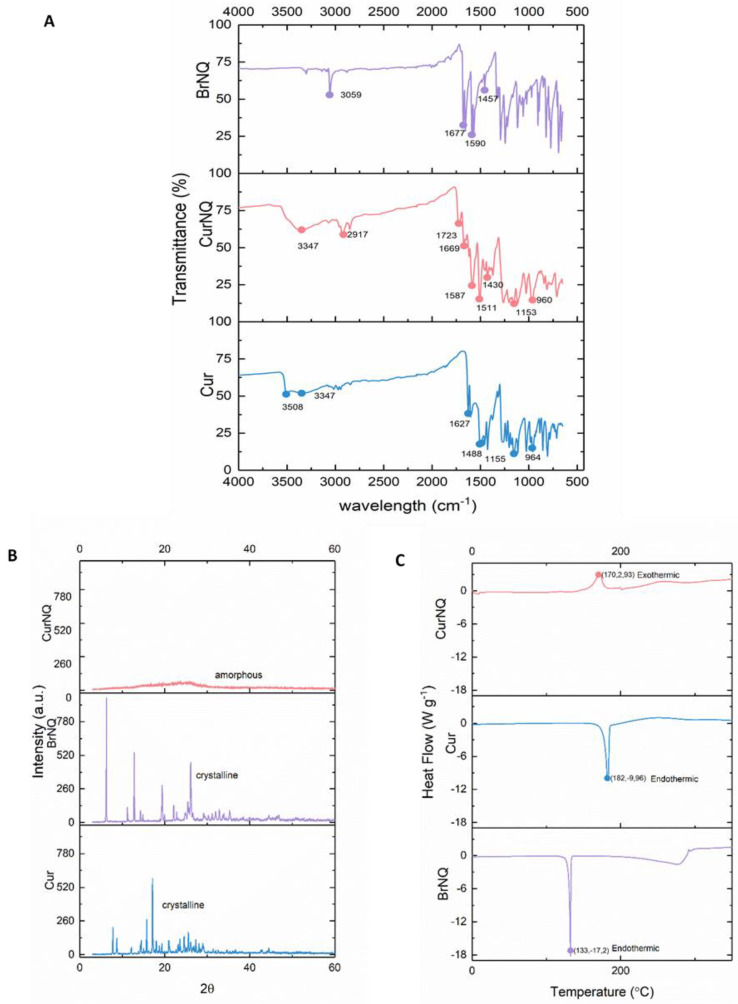
Structural characterization of CurNQ and comparison with curcumin (CUR) and BrNQ (**A**) FTIR spectra (**B**) powder X-ray diffraction (PXRD)—spectra. (**C**) Differential scanning calorimetry (DSC) thermograms. The spectral characteristics of CurNQ were elucidated through FTIR, DSC and PXRD and these spectral characteristics were compared to those of CUR and BrNQ.

In the FTIR spectrum of CUR (Figure 3A), the phenolic O-H stretching molecular vibration was observed around 3508 cm^−1^ [43]. In comparison in the FTIR spectrum of CurNQ, this absorption band was not observed, and this was indicative of the removal of this hydroxyl group, and the occurrence of a reaction at this site. A stretching vibrational band of the hydroxyl group of the keto-enol tautomerism (C2, 2′) is present at 3347 cm^−1^ and is visible in both CUR and CurNQ spectra. The alkyl ether groups (C11, 11′) of CUR are represented at wavenumber of 1155 cm^−1^, which is slightly shifted relative to the spectrum of CurNQ spectra (1153 cm^−1^). [44]. The aromatic ring of CUR and CurNQ (5,5′–10,10′) are assigned to vibrational bands 1488 and 1511 cm^−1^, respectively. The sharp band at 2917 cm^−1^ in CurNQ and 3059 cm^−1^ in BrNQ are representative of aliphatic and aromatic C-H groups. The carbonyl functional groups of BrNQ (C14, 14′, 21, 21′) were present at 1677 cm^−1^ in the BrNQ spectrum and 1669 cm^−1^ in the CurNQ spectrum. Moreover, the sharper peaks at 1590 and 1587 cm^−1^ and the small peaks present at 1457 and 1430 cm^−1^ are attributed to the aromatic C=C groups in BrNQ and CurNQ, respectively (C15–20, 15′–20′) [45]. The presence of the carbonyl groups and the aromatic rings in CurNQ indicate the successful synthesis of the desired molecule, as BrNQ functional groups are present in CurNQ. Thus, the key functional groups of Cur and BrNQ are present in the FTIR spectrum of CurNQ. 

The thermal properties of CUR, BrNQ and CurNQ were measured from 10–350 °C under inert conditions with a nitrogen flow rate of 50 mL·min^−1^ and with the temperature ramping at a rate of 10 °C per minute. DSC thermograms of CUR, BrNQ and CurNQ were obtained and are presented in Figure 3C. CUR and BrNQ display sharp endothermic peaks at 182 °C and 131 °C, respectively. These endothermic peaks represent the melting point of crystalline materials. Moreover, the peaks of these two endotherms agree with previously published data on the melting temperatures of both CUR and BrNQ, respectively [46,47]. Moreover, the presence of a single peak as well as the sharpness of the peaks indicates CUR and BrNQ’s high degree of purity. 

The exothermic peak present in the CurNQ thermogram at 172 °C is representative of a crystallization peak, this crystallization peak is shortly followed by an endothermic peak at 200 °C which represents crystal melting. The presence of the exothermic crystallization peak further concurs with the PXRD results, which indicate that CurNQ is an amorphous material. This release of energy represented by the exothermic peak is indicative of the ordering of the molecules to form a crystal which results in a release of energy as the material enters a more ordered state. CurNQ requires a temperature of 172 °C to convert it from an amorphous material into a crystalline solid. 

Powder X-ray diffraction (PXRD) was then performed on the three samples to ascertain the crystalline phases present in each sample and is presented in Figure 3. A change in crystalline phases indicates a unit cell transformation and thus a structural change. The PXRD of CurNQ takes the shape of a 90% amorph, in contrast to the highly crystalline structure of both CUR and BrNQ. This change to an amorphous nature is also verified by the broad exothermic peak of CurNQ in the DSC thermogram. 

Overcoming low aqueous solubility has become a fundamental issue in drug discovery, as many new chemical entities are hydrophobic and display low aqueous solubility [48]. Multiple approaches to improve a drug’s solubility exist, one of which is converting the crystalline drug to its amorphous form. Amorphous drug formulations have multiple favorable properties over crystalline formulations, the most important of which is increased solubility and increased bioavailability. The amorphous state is the highest energy form of a solid material with no long-range molecular order. Amorphous materials have larger molecular motions and more favorable thermodynamic properties compared to their crystalline counterparts owing to their high energy form [48]. Together, this results in amorphous materials displaying greater aqueous solubility thereby increasing bioavailability. Consequently, many researchers convert crystalline drugs into amorphous formulations to harness these favorable properties [49]. As evident in Figure 3, both CUR and BrNQ have high levels of crystallinity. On the contrary, CurNQ has a highly amorphous structure. The amorphous nature of CurNQ is confirmed through both the PXRD and the DSC spectra. Concerns exist relating to the re-crystallization of an amorphous formulation which would then decrease its solubility and bioavailability [50]. As seen in Figure 3, the crystallization peak of CurNQ occurs at 172 °C. This temperature would not be reached in vivo and thus removes the possibility of crystallization subsequent to treatment or during storage. The naturally amorphous nature of CurNQ is highly favorable and we expect this amorphous nature will enhance CurNQ’s bioavailability and further eludes to CurNQ being a highly promising drug candidate. 

The rationale for our novel theranostic molecule was to synthesize a novel molecule that exhibits fluorescent and anticancer properties, along with cancer targeting abilities. CurNQ contains multiple aromatic groups. Aromatic groups are known to exhibit intense fluorescence [51]. Consequently, CurNQ was expected to exhibit intense fluorescence owing to its highly aromatic chemical structure. This property can be exploited for cancer diagnostics, as a fluorescent signal can be detected and tracked. The excitation and emission spectra of a 1.5 µM solution of CurNQ was ascertained using the Shimadzu RF-6000 spectrofluorophotometer. The excitation peak of CurNQ was determined to be at 595 nm with the emission peak at 670 nm. A high intensity peak was detected, thus confirming the fluorescent nature of CurNQ and this fluorescent signal was further confirmed on the fluorescent microscope. Moreover, CurNQ displayed a large Stokes shift of 75 nm. A large Stokes shift is crucial for in vivo imaging applications as it reduces the overlap between the excitation and emission spectra thus reducing self-absorbance [52]. 

**Figure 4:**
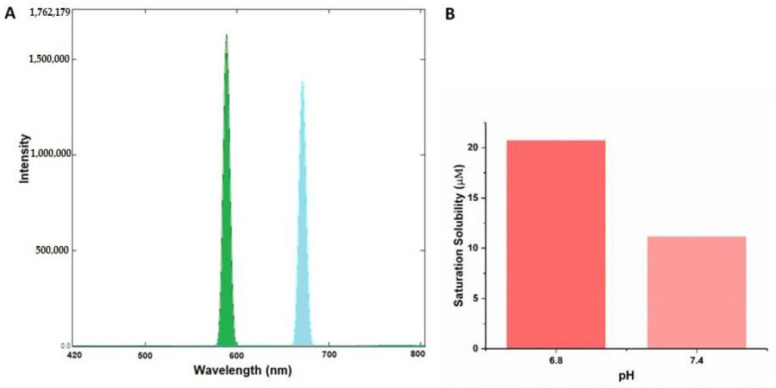
Fluorescence properties and pH specific properties of CurNQ. (**A**) Excitation and emission spectra of 1.5 µM solution of CurNQ. (**B**) Saturation solubility of a I.5 µM solution of CurNQ at pH 7.4 and 6.8.

The non-specificity and untargeted nature of current chemotherapeutics are major hindering factors to successful treatment, as unspecific treatment results in severe side effects and often displays poor efficacy. Consequently, cancer targeting and specific release of an active agent at the tumor location is essential. Multiple tactics can be employed to achieve this. Herein, we exploited the acidification of the tumor microenvironment that accompanies malignant transformation for targeted detection and treatment. As seen in Figure 4, CurNQ displays a low solubility at physiological pH (pH 7.4); with a small shift in pH from 7.4 to 6.8, CurNQ experiences a dramatic increase in solubility. The saturation solubility increased from 11.15 µM at pH 7.4 to 20.7 µM at pH 6.8. Hence, the saturation solubility almost doubled in response to this small change in pH. This establishes that CurNQ is highly pH responsive and demonstrates a pH-specific solubility. The slight acidification of the microenvironment surrounding a tumor is a hallmark feature of cancer and is termed the Warburg effect [14]. Therefore, CurNQ can detect this change in pH associated with malignant transformation resulting from its pH specific solubility. More than this, CurNQ displays bright fluorescence once soluble (Figure 4); thus, the detection of the acidic microenvironment will be accompanied by a sharp increase in fluorescent intensity. Indeed, CurNQ demonstrates cancer targeting and detection abilities owing to its pH specificity and fluorescent properties.

High content imaging is an advanced technique that combines high throughput techniques and fluorescent microscopy to obtain quantitative data from complex biological systems [53]. It is a robust method utilizing an automated microscope that allows for rapid, high content image acquisition and analysis [54]. Multiple independent measurements can be collected from a single cell or a single cell population. High content imaging removes the limitations of other techniques which collect a single average value for a population of cells, this technique allows for individual cells to be monitored and the effects of a drug to be quantified independently and simultaneously. Furthermore, high content imaging allows for the full effect of a selected compound to be evaluated in vitro [53]. Herein, this advanced technique was employed and using this technique, we were able to study the toxicity and dose dependency of CurNQ, CUR and BrNQ on three cell lines at 24-h intervals. Moreover, the morphological changes associated with treatment were quantified and analyzed. 

**Figure 5:**
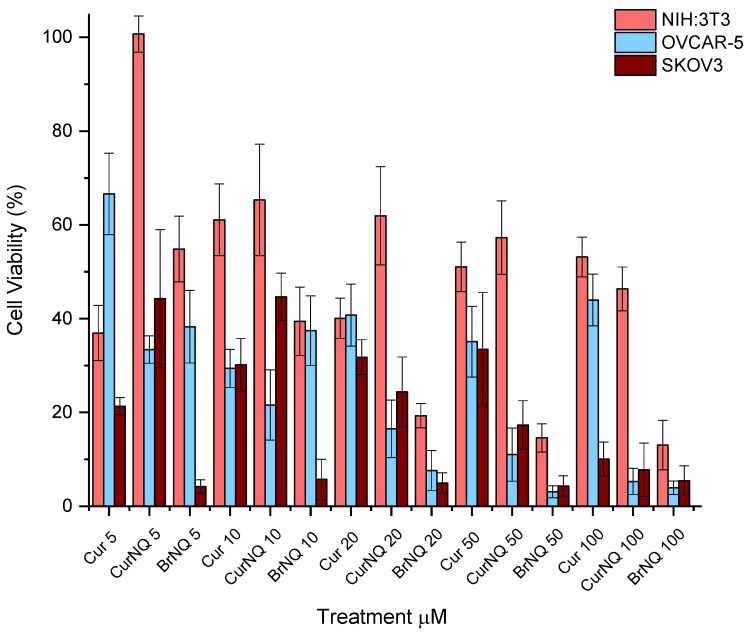
Cell viability assays were performed using a High Content Imaging System. Three cell lines were utilized, 2 ovarian cancer cell lines namely OVCAR-5 and SKOV3 and 1 healthy fibroblast cell line namely 3T3. Each cell line was treated with varying concentrations of CUR, CurNQ and BrNQ and treatments where incubated for a 24-h interval.

OC was selected as the model cancer type for this study. To this end, two ovarian cancer cell lines namely, OVCAR-5 and SKOV3 were utilized. The toxicity of CurNQ as well as any morphological changes that were induced through CurNQ treatment were ascertained using a high content imaging system. This high throughput imaging and screening method allows for an in-depth analysis of the effect CurNQ has on OC cells. The cells were also treated with equivalent concentrations of CUR and BrNQ. This acted as a comparative analysis, as previous studies have demonstrated the anticancer properties of these two compounds. Owing to the retention of active moieties of CUR and BrNQ in the novel molecule, it was expected that the viability results would resemble that of CUR and BrNQ; thus, all assays were performed with all three compounds. All three compounds displayed considerable reductions in cell viability in both OVCAR-5 and SKOV3 cell lines. OVCAR-5 cell viability was greatly reduced in response to CurNQ treatment. Low concentrations of CurNQ reduced cell viability to below 40% after 24-h incubation and a cell viability reduction occurred in a dose-dependent manner. The cell viability assay demonstrated that the toxicity of CurNQ to OVCAR-5 cells was in between that of CUR and BrNQ (Figure 5).

Additionally, the reduction in cell viability in response to CUR treatment was not dose dependent, but an overall general decrease in cell viability was observed. This may be owing to CUR degradation in cell culture media [32]. BrNQ treatment exhibited a far more potent cytotoxic effect than CUR treatment to OVCAR-5 cells (*p* = 0.000). In addition, the cytotoxic nature of BrNQ is highly dose dependent. A 20 µM treatment of BrNQ resulted in cell viability being reduced to below 10% after 24 h. Reductions in cell viability to CurNQ treatment were observed in both OVCAR-5 and SKOV3, and no statistical difference was observed between these two cell lines (*p* = 0.743). This sharp reduction in cell viability observed in response to CurNQ treatment in both OVCAR-5 and SKOV3 cell lines is promising data suggesting that CurNQ could act as a potent ovarian anticancer drug.

The above assay was also performed on the healthy fibroblast NIH:3T3 cell line. This acted as a control cell line and allowed for the effects of CurNQ on non-malignant cells to be ascertained. Traditional chemotherapeutics result in severe side effects owing to their non-specific toxicity, resulting in the death of healthy and malignant cells. The development of a treatment that is only toxic to cancerous cells would be highly advantageous and greatly enhance the clinical applications of such an anticancer agent. A comparison of the half maximal inhibitory concentration (IC_50_) values of each compound to each cell line is presented in Table 1. The IC_50_ values clearly demonstrate the selective toxicity of CurNQ towards ovarian cancer cell lines. NIH:3T3 cells did see a reduction in cell viability in response to CurNQ and BrNQ treatment. BrNQ displayed severe toxicity to the healthy cells with an IC_50_ value of 6.913 µM and cell viability dropped to below 15%, thereby further demonstrating its clinical barriers to use as an anticancer agent. On the contrary, NIH:3T3 cells did not experience severe toxicity to CurNQ treatment, with a non-potent IC_50_ value of 65.48 µM. Even at treatment concentrations of 100 µM CurNQ, the cell viability only dropped to 44%; although the reduction in cell viability was significant, it was far less severe than in response to BrNQ. These results are promising and indicate a low overall toxicity of CurNQ to healthy cells. This would further accentuate CurNQ’s application as a cancer treatment as healthy cells would be affected to a far lesser extent than malignant cells. 

A three-way ANOVA statistical analysis was performed with the above data using Stata/IC 15.1 software. Across the three cell lines, there was a statistically significant difference (*p* = 0.000) in Cur, CurNQ and BrNQ treatment for cell viability and the administered concentration was also significant (*p* = 0.0095). Additionally, the cell line had a significant impact on the treatment outcome (*p* = 0.0000). Tukey’s post-hoc test revealed that there was no statistical difference in the reduction in cell viability between OVCAR-5 and SKOV3 cell lines (*p* = 0.743); however, both cell lines’ cell viability was reduced by a significant difference compared to NIH:3T3 (*p* = 0.001; 0.000). These statistics demonstrate that CurNQ is more effective in malignant cell lines than in a healthy fibroblast cell line. This in conjunction with the IC_50_ values demonstrating CurNQ to offer selective toxicity to ovarian cancer cells. 

It has been well established that CUR displays poor solubility and bioavailability and is highly unstable. CUR degrades rapidly in various conditions and has multiple confirmed metabolites. Wang et al. [32] demonstrated that 90% of CUR degrades within 30 min in phosphate buffer saline (PBS) as well as a 90% degradation in cell culture media supplemented with fetal bovine serum (FBS) after 8 h. This rapid and complete degradation of CUR in PBS and cell culture media can explain the absence of a dose-dependent cell viability response to CUR treatment. All treatments were prepared in PBS and then added into the cell culture media. This would have resulted in the total degradation of CUR prior to the 24-h incubation period. CUR degrades into at least five different metabolites, some of which are thought to have anticancer properties of their own and some of which do not [33]. The unpredictability of CUR degradation precludes the results obtained to be highly accurate as each treatment event could have resulted in the formation of different metabolites as well as at varying proportions. This lack of stability and rapid degradation of CUR in aqueous solutions is greatly overlooked and is not considered in majority of research articles utilizing CUR alone. These prohibitive properties preclude CUR’s use as an anticancer agent.

**Figure 6:**
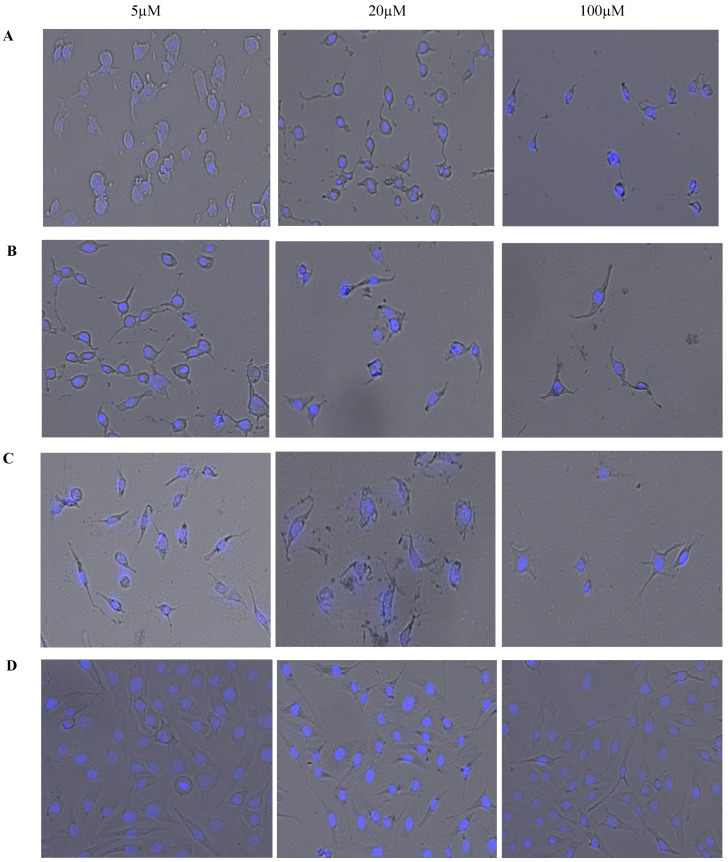
A visual representation of the dosage effects of (**A**) CUR, (**B**) CurNQ (**C**) BrNQ and (**D**) PBS treatment on OVCAR-5 cells after a 24-h incubation period. Brightfield microscopy images are overlaid on fluorescent microscopy images.

Figure 6 displays brightfield images overlaid on fluorescent images with 4′,6-diamidino-2-phenylindole (DAPI) staining. These images were acquired simultaneously using high content imaging. This combination of brightfield and fluorescent microscopy allows for a visual representation of the effect of treatments on OVCAR-5 cells’ morphology after 24 h. Phenotypic changes that occurred in response to treatment can be visually monitored and qualitative data can be obtained. The reduction in cell count in response to an increased dosage of each treatment is clearly apparent and Figure 6 is a visual representation of the cell viability data displayed in Figure 5. As seen in Figure 6, cellular morphology becomes less consistent and variations in cell morphology are evident with increasing concentrations of CurNQ. Importantly, the high content imaging software that was used to acquire these images also gives quantitative data on the morphology of the cells in these images, allowing for changes in morphology to be quantitatively assessed. This combination of qualitative and quantitative data makes high content imaging a powerful tool for drug discovery. The phenotypic changes in response to treatment can elucidate important cellular events and further reveal the mechanisms of action and effectiveness of a target compound. The use of machine learning to evaluate the morphological changes in response to a test compound is an advanced method to analyze the potential effectiveness of a novel compound. The cell morphology of OVCAR-5 cells in response to varying concentrations of CUR, CurNQ and BrNQ was quantified using the CELENA^®^ X High Content Imaging System and their algorithm. 

**Figure 7:**
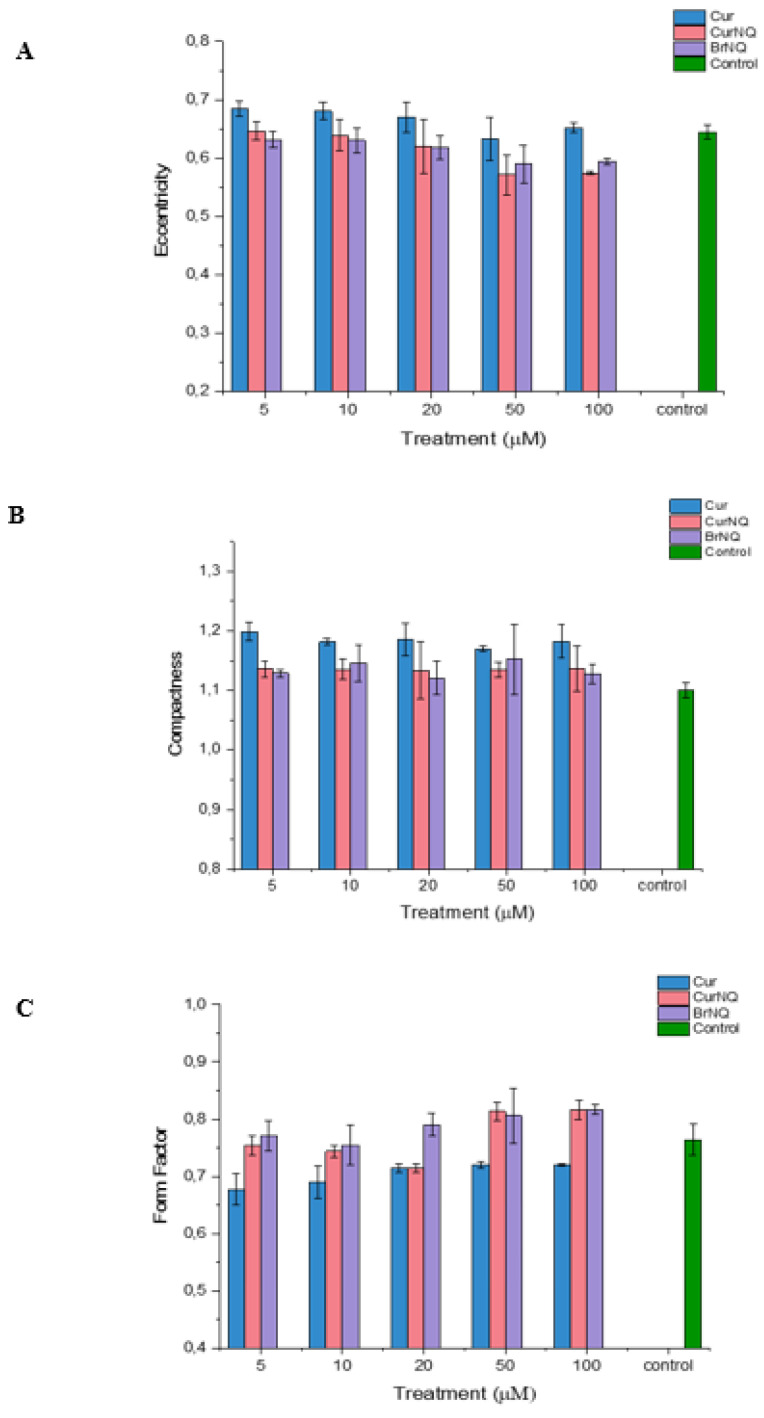
Cell morphology analysis of OVCAR-5 cells subsequent to CUR, CurNQ and BrNQ treatment. Cell morphology data obtained on the CELENA^®^ X. (**A**) Eccentricity. (**B**) Compactness. (**C**) Form factor.

Apoptosis is characterized by chromatin condensation, the disassembly of nuclear scaffold proteins and DNA fragmentation [55]. These events lead to the condensing of the nucleus, thereby resulting in the nucleus to become more rounded. This rounding of the nucleus can be detected, and the extent of rounding can be quantified using high content imaging, consequently allowing for apoptosis to be detected and quantified. Compactness is a measure of a spheroid’s fullness. An equally filled circle will have a compactness of one, with irregular objects or objects with holes having a value greater than one. Therefore, in the context of cellular morphology, we would expect the compactness of healthy cells to be close to one, and cells that are experiencing stress and that are undergoing apoptosis to have a compactness exceeding one. Healthy cells would exhibit a compactness value close to one as a healthy nucleus is evenly “filled” with DNA. Apoptosis induces DNA fragmentation, thus reducing the amount of DNA evenly spread out across the area of the nucleus; thus, a cell undergoing apoptosis would have a compactness exceeding one as DNA fragmentation would make the nucleus appear to have ‘holes’ or to appear irregular. Our results concur with these assumptions. The compactness of all treated cells is greater than the control (Figure 7). This indicates that DNA fragmentation and nuclear distortion is occurring as the nuclei become irregular subsequent to treatment. This concurs with the results displayed in Figure 5 that demonstrate a substantial reduction in cell viability in response to treatment. 

Moreover, the form factor and eccentricity values were obtained. These values indicate the level of circularity of an object. Form factor is calculated as 4 × π × Area/Perimeter2 and equals one for a perfectly circular object. The eccentricity is the ratio of the distance between the foci of the ellipse and its major axis length. The value is between zero and one. An ellipse with an eccentricity of zero is a circle, while an ellipse with an eccentricity of one is a straight line. Chromatin condensation and DNA fragmentation is a hallmark of apoptosis [55]. Chromatin condensation causes the DNA to become dense, thereby condensing the nucleus into a more circular object. Therefore, a cell undergoing apoptosis would display a higher form factor and lower eccentricity. 

As evident in Figure 7, the form factor is only greatly altered in cells treated with CurNQ and BrNQ greater than 50 µM, and this treatment induced a large increase in the form factor. Therefore, after 50 µM of CurNQ or BrNQ treatment, higher levels of apoptosis would be expected as chromatin condensation causes the nucleus to become more rounded. Indeed, this aligns with the cell viability results in Figure 5. Moreover, eccentricity decreased in a dose-dependent manner in response to CurNQ treatment, and it was reduced to a greater extent than both CUR and BrNQ-treated cells. A lower eccentricity value indicates the nucleus is becoming more rounded, thus implying that chromatin condensation and apoptosis are occurring. All three indicators of cell morphology align in indicating that apoptosis is induced in response to CUR, CurNQ and BrNQ treatment. The nuclear morphology is altered, the nuclei becomes more irregular and circular in shape. This eludes to chromatin condensation and DNA fragmentation, thus indicating the induction of apoptosis.

**Figure 8:**
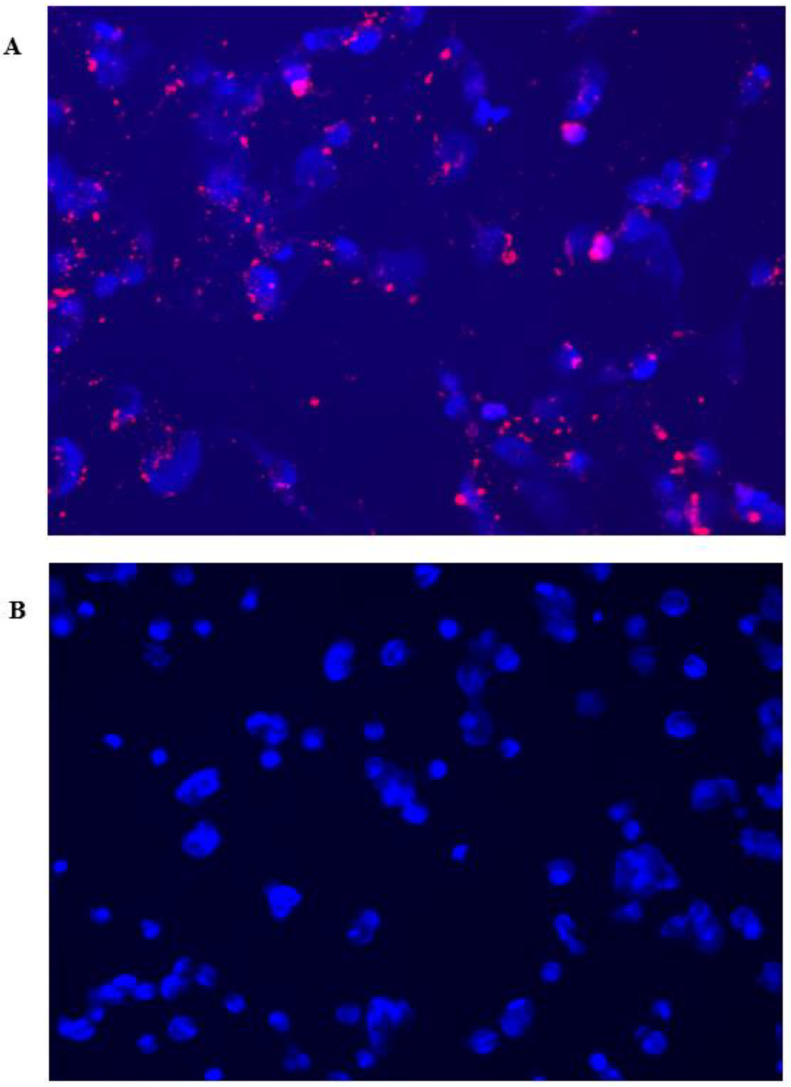
Fluorescent microscopy images of (**A**) CurNQ loaded onto mesoporous silica nanoparticles. (**B**) Control sample where cells were treated with unloaded mesoporous silica nanoparticles. Nuclei were stained with DAPI.

To ascertain the fluorescent detection capabilities of CurNQ, CurNQ was loaded onto mesoporous silica nanoparticles (MSN)**.** MSN were synthesized according to a previously established protocol [56] and OVCAR-5 cells were treated with the loaded nanoparticles for 24 h, thereby allowing for uptake to occur. The loading of CurNQ onto a nanosystem allowed for its in vivo fluorescent and potential detection applications to be evaluated using the fluorescent microscope on the high content imaging system. As presented in Figure 8, the loaded nanoparticles displayed clear, distinctive, high-intensity fluorescence. Moreover, a high target to background ratio was observed with a low background or unspecific fluorescence. These favorable fluorescent properties indicate that CurNQ may facilitate tumor detection applications.

## 3. Materials and Methods 

### 3.1. Materials

Curcumin (CUR) (95%) was received ex Gratia from Jiaherb Phytochem (Xian, China). 2-Bromo-1,4-naphthoquinone, 98%, dimethylformamide (DMF), DAPI, and sodium hydroxide pellets were procured from Sigma-Aldrich (St. Louis, MO, USA) and potassium carbonate was purchased from ACE chemicals (Johannesburg, South Africa). All other solvents, including methanol, ethanol, ethyl acetate, hexane and salts were of analytical grade and were used as received. Silica Gel 60 (0.015–0.040 mm) was purchased from Merck and thin-layer chromatography plates were purchased from Macherey-Nagel (Duren, Germany) and were viewed under UV light (254 nm and 366 nm). Cell lines were purchased from Cell Biolabs, Inc. (San Diego, CA, USA) and Fox Chase Cancer Facility (Philadelphia, PA, USA). Cell culture consumables, including growth media and reagents, were purchased from Thermo Fischer Scientific (Waltham, MA, USA). 

### 3.2. Methods

#### 3.2.1. Synthesis of Novel Cur Derivative CurNQ

The hydroxy moiety of lawsone was replaced with a reactive bromine group allowing for reactivity at this moiety. For the purpose of ease, the lawsone derivative 2-Bromo-1,4-naphthoquinone (BrNQ) was purchased and utilized as received. In a fume hood shielded from light, one equivalent of CUR (50 mg, 1.36 mmols) was reacted with two equivalents of BrNQ (64 mg, 2.72 mmols) and two equivalents of potassium carbonate (37.6 mg, 2.72 mmols) in dry DMF (5 mL) as depicted in Figure 1. The reaction was monitored continuously by thin-layer chromatography (TLC) and, after 2 h, the reaction was complete. The resulting reaction was quenched with dilute Hydrochloric acid (HCL) solution and washed three times with ethyl acetate and water. The organic phase was collected, dried over anhydrous magnesium sulphate, filtered, and the organic solvent was removed under reduced pressure to obtain a crude product. The pure product was isolated via silica column chromatography using 30% *v*/*v* ethyl acetate:hexane eluent. The combined fractions were subjected to solvent evaporation and the resulting purified product, CurNQ, was dried in vacuo and analyzed.

#### 3.2.2. Nuclear Magnetic Resonance—Structural Determination

Multinuclear 1D, ^1^H and ^13^C NMR spectroscopy were performed to elucidate the structure of the synthesized molecule. ^1^H and ^13^C NMR spectroscopy were performed on the Bruker AVANCE II 500 MHz (Bruker Biospin, Ettlingen, Germany) using deuterated dimethyl sulfoxide (d-DMSO) as a solvent at room temperature. All chemical shift values are reported in parts per million and coupling constants (J-values).

#### 3.2.3. High Performance Liquid Chromatography–Mass Spectrometry—Structural Confirmation 

The analysis of CurNQ was performed on a Bruker Compact electrospray ionization (ESI) Q-TOF high resolution compact mass spectrophotometer. 10 μL of the sample was injected into the HPLC and run through the C18 column (5–95% ACN). The solvent system was made up of solvent A, consisting of 0.1% formic acid in H_2_O (% *v*/*v*) and 50% solvent B, consisting of 0.1% formic acid in acetonitrile (% *v*/*v*). The samples were diluted to a concentration of approximately 10 ppm in a mixture of methanol and formic acid (1:1). The mass spectrum analysis was processed using the Bruker Daltonics data analysis program.

#### 3.2.4. Fourier Transform Infrared Spectroscopy (FTIR)—Characterization of Structural Moieties 

FTIR was performed using a single-reflection diamond detector on a PerkinElmer Spectrum 100, (PerkinElmer, Llantrisant, Wales, UK) to analyze the vibrational changes in the chemical structures which occurred during product formation. Through obtaining the spectra of CUR, BrNQ and CurNQ, the chemical structure of the product could be ascertained, as FTIR allows for functional group identification. All samples were scanned from 4000–600 cm^−1^ at room temperature at a constant pressure of 120 psi. 

#### 3.2.5. Differential Scanning Calorimetry (DSC)—Characterization of Thermal Properties 

Differential scanning calorimetry (DSC) was performed on the Mettler Toledo DSC-1 STARe system (Mettler Toledo, DSC1, STARe System, Swchwerzenback, Switzerland) to evaluate the heat flow required for phase transition and thermal properties of the new molecule as well as both starting materials. 6mg of each sample was loaded and sealed into a 40 mL aluminum crucible pan and a small puncture was inserted into the top of the crucible and was heated at a rate of 10 °C/min from 10–350 °C under constant N_2_ gas. The instrument was heat- and enthalpically calibrated using indium metal (99.99%).

#### 3.2.6. Powder X-ray Diffraction (PXRD)—Crystallinity Characterization

Powder X-ray diffraction (PXRD), utilizing a variable and fixed slit system, was used to determine the crystallinity and atomic spacing and unit cell dimensions of the new molecule. This characterization technique was performed on the Rigaku MiniFlex 600 Benchtop X-ray Diffractometer (Rigaku Corporation, Tokyo, Japan). A powdered sample was packed onto the sample holder and was analyzed at a scanning rate of 15° per minute at a diffraction angle range of 3°–90° with a degree step of 0.02, a voltage of 40 kV and a current of 15 mA. 

#### 3.2.7. Fluorescent and Absorbance—Spectrofluorometric Characterization

The fluorescent and absorbance properties of CurNQ were evaluated to ascertain the fluorescent nature of CurNQ. The fluorescent properties of CurNQ were determined through obtaining the absorption and fluorescence spectra which were measured and recorded on the Shimadzu UV-1800 spectrophotometer and Shimadzu RF-6000 spectrofluorophotometer (Shimadzu Scientific Instruments, Columbia, MD, USA), respectively. 

#### 3.2.8. Cell Culture 

The ovarian carcinoma cancer cell lines OVCAR-5 and SKOV3 were purchased from Fox Chase Cancer Facility, USA, Jerkitown, CA. The healthy fibroblast cell line NIH: 3T3 was utilized as a control. The cells were cultured in DMEM with the media being supplemented with 10% fetal bovine serum and 1% penicillin-streptomycin. Cells were incubated at 37 °C with 5% CO_2_. Upon the cells reaching 70–80% confluency, the cells were washed, and sub-cultured by harvesting using trypsin/EDTA. 

#### 3.2.9. High Content Imaging—Evaluation of Cellular Cytotoxicity

Cells were grown until 80% confluency prior to utilization. Upon confluency, media was removed, and cells were washed twice in sterile PBS. Trypsin/EDTA was then added to the flasks and incubated at 37 °C for 5 min, allowing the cells to detach from the bottom of the flask. These cells were then collected and centrifuged. The pellet was resuspended, and the cells were counted using the trypan blue exclusion assay. A predetermined and equal number of cells were then added into each well of a 96-well plate. The cells were incubated overnight at 37 °C and at 5% CO_2_, allowing the cells to attach to the plates. The following morning, cells were treated with each sample (CurNQ, CUR and BrNQ) at concentrations of 5 µM, 10 µM, 20 µM, 50 µM and 100 µM. Each treatment was performed in quintuplicate. Treatments occurred for 24 h. Subsequent to this time period, media was removed, and cells were fixed with 5% formaldehyde in PBS for 20 min. Next, cells were incubated for 15 min in a 1 µg/mL solution of DAPI. The plates were then washed several times with PBS to remove all residual DAPI and to reduce background fluorescence. All plates were stored in the dark. Plates were then run on the Logos Biosystem CELENA^®^ X High Content Imaging System and analyses were performed using their proprietary software. IC_50_ values were calculated using a Prism Graph. Using the Logos Biosystem software, a pipeline was set up to determine the nuclei count, form factor, compactness and eccentricity. The following definitions were used:

Eccentricity = ratio of the distance between the foci of the ellipse and its major axis length.

Compactness = mean squared distance of the object’s pixels from the centroid divided by the area.

Form Factor = 4 × π × Area/Perimeter2

#### 3.2.10. Statistical Analysis

All experiments were performed in triplicate or quadruplet for statistical purchases and standard deviations were calculated. One-way ANOVA, three-way ANOVA and Tukey’s test for post-hoc analysis were performed on acquired data. Statistical analyses were all performed on Stata/IC15.1. 

## 4. Conclusions

A novel theranostic molecule (termed CurNQ) with anticancer targeting properties was synthesized through a Williamson ether synthesis reaction between the phytochemicals CUR and lawsone. The CurNQ structure was confirmed through ^1^H and ^13^C NMR and HRMS-ESI. CurNQ was found to be highly pH specific with preferential solubility at the pH of the tumor microenvironment, enabling tumor targeting in OC. Moreover, CurNQ facilitated tumor detection once soluble in the tumor microenvironment due to its intense fluorescence. CurNQ was also cytotoxic to two OC cell lines, thereby extending its application as a theranostic system. 

## Figures and Tables

**Table 1:** IC_50_ values of each compound against two ovarian cancer cell lines and a healthy fibroblast cell line.

Cell Line	Cur IC_50_ (µM)	CurNQ IC_50_ (µM)	BrNQ IC_50_ (µM)
OVCAR-5	9.638	4.048	4.854
SKOV3	4.791	5.354	2.738
NIH:3T3	43.93	65.48	6.913
